# Deciphering the M-cell niche: insights from mouse models on how microfold cells “know” where they are needed

**DOI:** 10.3389/fimmu.2024.1400739

**Published:** 2024-05-28

**Authors:** Diana Del Castillo, David D. Lo

**Affiliations:** Division of Biomedical Sciences, School of Medicine, University of California, Riverside, Riverside, CA, United States

**Keywords:** microfold, transcytosis, mucosal immunity, immune surveillance, epithelium

## Abstract

Known for their distinct antigen-sampling abilities, microfold cells, or M cells, have been well characterized in the gut and other mucosa including the lungs and nasal-associated lymphoid tissue (NALT). More recently, however, they have been identified in tissues where they were not initially suspected to reside, which raises the following question: what external and internal factors dictate differentiation toward this specific role? In this discussion, we will focus on murine studies to determine how these cells are identified (e.g., markers and function) and ask the broader question of factors triggering M-cell localization and patterning. Then, through the consideration of unconventional M cells, which include villous M cells, Type II taste cells, and medullary thymic epithelial M cells (microfold mTECs), we will establish the M cell as not just a player in mucosal immunity but as a versatile niche cell that adapts to its home tissue. To this end, we will consider the lymphoid structure relationship and apical stimuli to better discuss how the differing cellular programming and the physical environment within each tissue yield these cells and their unique organization. Thus, by exploring this constellation of M cells, we hope to better understand the multifaceted nature of this cell in its different anatomical locales.

## 1 Introduction

Existing as a boundary between the body and the internal, topological “outside”, the mucosa creates a landscape for a complex dynamic between the luminal contents and the body itself. Whether it is the lung, the gut, or the eye, these tissues are exposed to an assortment of bacteria, viruses, and particulates and, on the microscopic level, create an interesting problem: what comes in and what stays out? Immunologically, this important interaction has implications on not only response but tolerance as well, and a series of systems are in play to perform the necessary role of surveillance.

Generally categorized as stratified or simple, the epithelium has methods of antigen acquisition once thought to be specific to each as they have different requirements in transferring particles between the lumen and subepithelia. An antigen trafficking mechanism thought to be specific to simple epithelia involved goblet cells. Goblet cell-associated antigen passages (GAPs) facilitate the transport of soluble antigens from the lumen to the lamina propria; however, they have only been noted in the simple columnar epithelium of the intestine (1–3). Dendritic cells (DCs), in contrast, seem to be more versatile at this junction, as they are able to extend their dendrites through the epithelium and directly sample luminal antigens (4); thus, this process has been reported in various tissues. In the intestinal mucosa, DCs in the intestinal lamina propria form transepithelial dendrites (TEDs) that sample luminal bacteria (5, 6). Further, in a human airway model and studies of patients with allergic rhinitis, results suggest that these cells can exhibit similar tendencies in the lung and nasal mucosae (7, 8). In the ocular mucosa even, DCs of the conjunctiva can extend across the epithelial barrier as a response to an increase in microbial colonization (9). Interestingly, they have also been described to employ similar machinery in non-mucosal tissues such as in the hair follicle and thymic endothelium (10, 11). Of the two antigen-capturing methods described, M cells appear to be as versatile as the latter, appearing in many tissues and epithelium types. However, tied to the epithelium and less motile than an antigen-presenting cell (APC), these cells require a complex set of triggers to recognize antigen-sampling needs and cue them into differentiating.

Originally discovered in the gut-associated lymphoid tissue (GALT) (12, 13), M cells have best been characterized in Peyer’s patches (PPs). Although once assumed to be restricted to the gut, they seem to follow a broader appearance pattern and have now been identified in the mucosa of the intestines, colon, nasal passages, lung, and eye as well as among the epithelia of the thymus. With the continued discovery of these cells in other, more far-flung, tissues, the issue of establishing an M-cell definition becomes increasingly crucial. This has been a topic of discussion since variations in *in vitro* models emerged, and many excellent publications have reviewed the molecular markers associated with M cells (14–19). M cells display many overlapping common markers and morphologic characteristics despite the remarkable range of disparate tissues and circumstances where they arise. Therefore, in this discussion, we aim to emphasize the environmental, physiologic, and physical (i.e., electrostatics and fluid dynamics) features that lead cells in disparate settings to develop a convergent phenotype. Ultimately, our interest is in how the functional needs give rise to this phenotype from different developmental precursors. Since the M-cell phenotype seems to converge identifiably (through a set of associated markers and morphology) and functionally (through uptake capabilities), we will further consider the local environmental conditions that dictate where they appear and how they pattern. Here, Peyer’s patch M cell is merely a starting point as a prototypic M cell, and through this lens, we will further explore M-cell appearances that extend outside of the conventional gut, lung, and nasopharyngeal mucosae. M cells of the eye, dorsal tongue, and thymus are more recently described in mice, and thus, we have limited information on them; however, we will use the information gathered from the prototypical Peyer’s patch M cell—and these non-classical cells—to describe and build upon M-cell definition and function in various tissues.

## 2 The quintessential M cell

Classically arising from Lgr5+ endodermal stem cells (20), the M cell develops a set of features that have become hallmarks including a growing list of genetic markers, functionally relevant morphology, and transcytotic capability. In exploring these attributes in the GALT-associated cell, we will explore the cellular and molecular characteristics used to define and identify the “prototypical” M cell.

### 2.1 Applying molecular definitions to track cell phenotypes

Previously relying on less specific methods such as lectin histochemistry (e.g., UEA1+ and WGA−), the identification of M cells has made significant strides with an increasingly comprehensive transcriptomic approach. Without cataloging the entirety of this list of markers, we will mention those that have provided insight into function or organization relevant to our discussion. Genes thought to be involved in the transcytotic ability of the cells include *Anxa5* (annexin A5) and *Marcksl1* (myristoylated alanine-rich protein kinase C substrate) (21) as well as *Tnfaip2* (TNF-α-induced protein 2), and M cell-specific chemokines Ccl20, Ccl6, and Ccl9 have played a significant role in their recognition (22, 23). Notably, two transcription factors have been implicated in key developmental pathways for these cells, SpiB and Sox8, where SpiB has been dubbed the closest to being an M-cell master regulator (24, 25). Still, only some recognition genes are SpiB-dependent such as *Ccl9*, *Tnfaip2*, and *Gp2* (26). In particular, glycoprotein 2 (GP2) has been shown to be highly expressed in functionally mature M (27) cells and is potentially the most common target for immunostaining studies, having been used in most of the tissues discussed (**Table 1**). More recently, however, GP2 immunostaining may prove to be not as specific or universal in all contexts (44, 50). *Pglyrp1*, which encodes for peptidoglycan recognition protein (PGRP-S), is a protein with bactericidal effects that is also upregulated in the follicle-associated epithelium (FAE) and further in M cells, at least in the mouse (23, 31, 51). Studies using a reporter mouse for M cells, in which a red reporter fluorescent protein (dsRed) is expressed under the control of the Pglyrp1 promoter, have used them to identify these cells in the GALT, nasal-associated lymphoid tissue (NALT), and thymus (48, 52).

**Table 1:** M-cell characterization by tissue.

Tissue	Confirmed identification markers	Morphology	Functional characterization	RANKL response	Lymphoid tissue	Seminal studies
**PP**	Lectin binding (UEA1) (28) Fucose moiety (NKM 16-2–4 mAb) (29) Gp2 (22) Spib (24) Tnfrsf11b (OPG) (30) Pglyrp1 (PGRP-S) (31) Ccl20 (22) Ccl9 (22) Sox8 (25) Tnfaip2 (32) Anxa5 (21) Marcksl1 (22) Sgne1 (23) Cklf (22)	Blunted apical membrane, basolateral pocket	Particle and bacterial transcytosis	+	+	(12) (33) (24) (22) (34)
**Villous M cell**	Lectin binding (UEA1+, WGA−) Fucose moiety (NKM 16-2–4 mAb) (29) Ccl9 (22) Ccl6 (22) Cklf (22)	Blunted apical membrane, basolateral pocket	Bacterial uptake	+	−	(35) (22)
**NALT**	Lectin binding (UEA1) (36) Gp2 (36, 37) Pglyrp1 (PGRP-S) (31) Spib (37) Ccl20 (36) Ccl9 (36, 37) Tnfaip2 (37)	Blunted apical membrane, basolateral pocket	Particle and bacterial transcytosis	+	+	(Rat, 38) (37) (36)
**Lung (BALT and trachea)**	Lectin binding (UEA1) (39) Gp2 (40, 41) Tnfaip2 (40, 41) SpiB (41) Ccl20 (41) Ccl9 (41) Marcksl1 (41) Sox8 (41)	Blunted apical membrane, basolateral pocket	Particle uptake	+	+(BALT) −(Trachea)	(39) (40)
**“Respiratory M cells”**	Lectin binding (UEA+, WGA−) (42) Fucose moiety (NKM 16-2–4 mAb) (42)	Blunted apical membrane, no basolateral pocket	Protein and bacterial uptake	?	−	(42)
**TALT**	Lectin binding (UEA1) (43) Fucose moiety (NKM 16-2–4 mAb) (43) Gp2 (44) Sox8 (44) Tnfaip2 (44) SpiB (44) Tnfrsf11b (OPG) (44)	Potential basolateral pocket	Particle uptake	+	+	(43) (44)
**CALT**	Lectin binding (UEA1+, WGA−) (45)	Blunted apical membrane	Bacterial uptake	?	+	(Guinea pig, 46) (45)
**Type 2 taste cells**	Gp2 (47) Marcksl1 (47) Ccl9 (47) Anxa5 (47) Sgne1 (47) Spib (47)	Apical projection extending from taste pore	Particle uptake	+	−	(47)
**Thymus**	Gp2 (48, 49) Tnfrsf11b (OPG) (48, 49) Pglyrp1 (PGRP-S) (48) Ccl6 (48, 49) Ccl9 (48, 49) Ccl20 (48, 49) Tnfaip2 (48, 49) Spib (48, 49) Sox8 (48, 49) Anxa5 (48) Marcksl1 (48)	Non-mucosal, irregular shape with no identifiable polarity	Particle uptake	+	+*	(49) (48)

This is not an exhaustive list of markers. Different methods (transcriptomics, immunostaining, etc.) were used and are available in the cited sources.

+, present; −, not present; ?, not studied; PP, Peyer’s patch; NALT, nasal-associated lymphoid tissue; BALT, bronchus-associated lymphoid tissue; TALT, tear duct-associated lymphoid tissue; CALT, conjunctiva-associated lymphoid tissue.

Gene (PROTEIN) when relevant.

+* Although technically associated with lymphoid tissue, microfold medullary thymic epithelium cells (mTECs) exist in the thymic medulla, which does not have the conventional organization of a secondary lymphoid tissue-like inducer site.

Still, the molecular identification of an individual cell can only tell us so much; taking a step back and analyzing the tissue-level organization yield insight into an intrinsic M-cell patterning. Characteristically, Peyer’s patch M cells show a radial spoke pattern across Peyer’s patch follicle epithelium when imaged “en face” and labeled with mature cell markers such as GP2 (or PGRP-S) and UEA1 (31, 53). This indicates the crypt-to-FAE differentiation pattern of these cells and illustrates the fact that these M cells’ origins are in the instructions provided to the crypt stem cells at the margins of Peyer’s patch, presumably through cytokines produced within the organized lymphoid tissue. Thus, the appearance and distribution of these M cells are determined by a more sophisticated communication between the lymphoid tissue and the crypt stem cells. This requires the crypt to only produce M cells on the side facing the follicle and also produce them in a coordinated manner to result in the distributed radial spoke pattern.

Likewise, the cell–cell communication involved in this patterning has only just been explored. *Tnfrsf11b*, a recognized marker upregulated in M cells, encodes for osteoprotegerin (OPG), which functions as a decoy receptor to pro-differentiation signaling (RANK-RANKL) (30). Similarly, Notch signaling has been shown to be upregulated in M cells and implicated in the regulation of M-cell numbers and distribution such that deletion of Notch1 in the intestine resulted in increased numbers and increased clustering (54, 55). As more targeted approaches have begun to establish the molecular fingerprint of these cells, it is clear that their differentiation is far more sophisticated than simply providing a simple switch to activate the M-cell phenotype. Disappointingly, too many discussions of intestinal crypt stem cells only mention the variety of differentiated phenotypes present among villus epithelium and deep crypt secretory cells, apparently oblivious to the rich complexity of M-cell induction.

**Figure 1:**
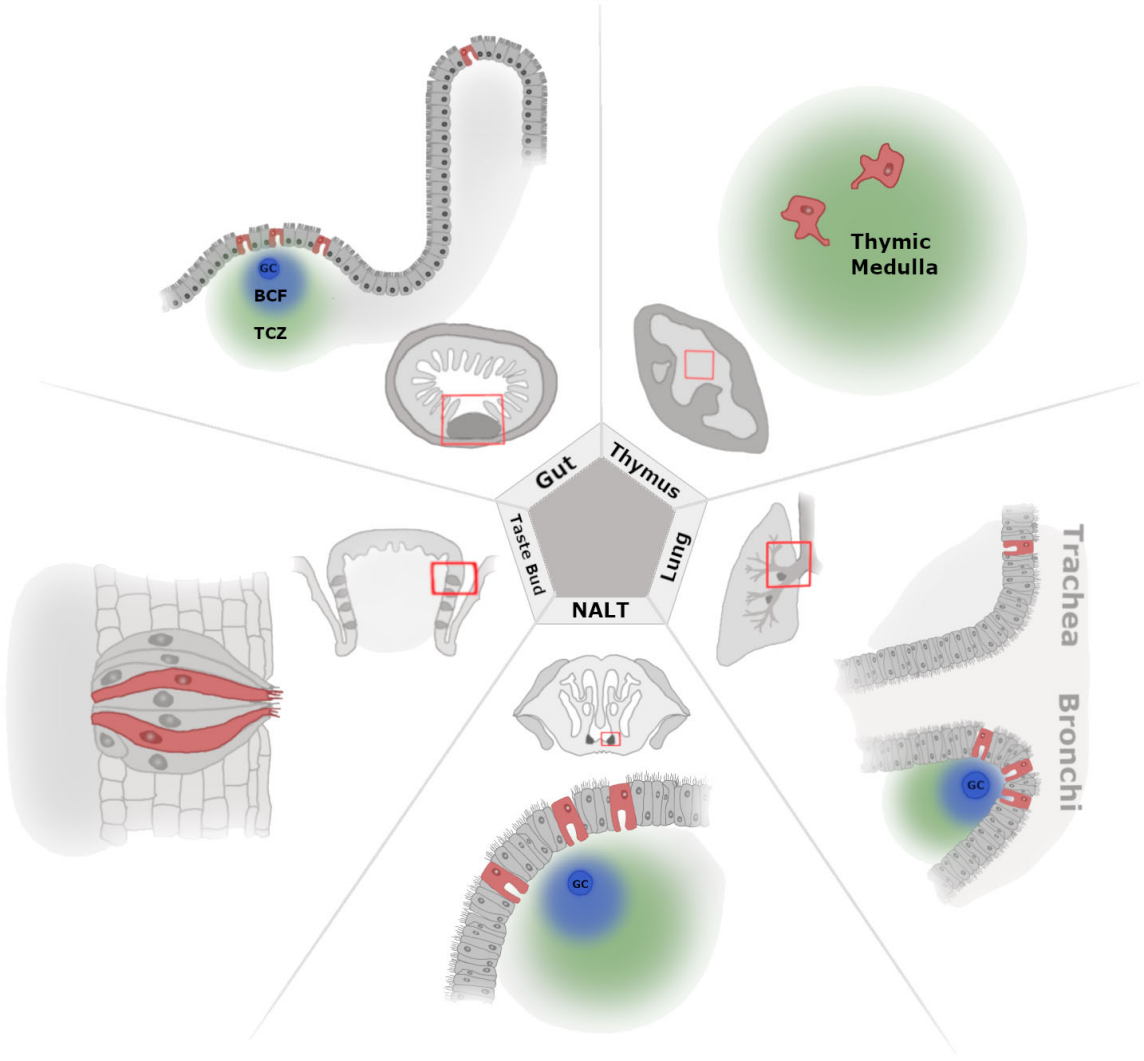
A zoomed-out view of tissue-level M-cell localization. Illustration of select tissues where M cells have been documented with cell level and zoomed-out perspectives. Beginning at the top left and moving clockwise, we show the M cells present in the gut [Peyer’s patch (PP) and villous M cells], thymus, lower airway lung [trachea and bronchus-associated lymphoid tissue (BALT)], upper airway [nasal-associated lymphoid tissue (NALT)], and taste bud (circumvallate papillae). In the zoomed-out images, the red boxes indicate where the cell-level image focuses within the larger anatomical location, and lymphoid tissue is shown as the darkest areas of the tissue, where applicable (PP, NALT, and BALT). For the thymus, the zoomed-out image shows the cortex and medulla as dark gray and light gray, respectively. Germinal center (GC), B-cell follicle (BCF; blue cloud), and T-cell zone (TCZ; green cloud).

While Peyer’s patch M-cell induction is complex enough, the appearance of M cells in a variety of other settings raises the question of how to generate a definitive M-cell molecular definition. While comparing characteristics (**Table 1**), the similarities become clearer as a set of reappearing molecular markers, uptake capabilities, RANKL response, and some extent of morphological adaptation. This becomes more striking despite differences such as tissue type and origin of progenitors, epithelium type, and association with lymphoid structures (**Figure 1**). This discussion underscores that this is only the start of M-cell identification, and additional information is needed to provide a roadmap of these cells at different developmental stages and tissue settings.

### 2.2 From morphological anomalies to specialized surveillance

The discovery of M cells was largely based on their distinct morphology. Under scanning and transmission electron microscopy, their apical and basolateral adaptations set them apart from the complex landscape of the mucosa-associated lymphoid tissue (MALT). Situated strategically in the FAE of Peyer’s patches, M cells were initially discovered and characterized based on these characteristics (12, 56). Apically, M cells show a departure from the typical organized brush border with short and irregular microvilli. Immunostaining of microvillar proteins, such as actin and villin, further aids in M-cell identification by highlighting the absence of staining due to their unique microvillar structure (57). On the basolateral end, the deeply invaginated membrane forms characteristic pockets. These features prove essential for efficient transcytosis at the mucosa.

The localization of these cells at this crucial barrier junction as well as their specialized appearance highlights the need for surveillance and the evolution of mechanisms to achieve this. The quintessential functional characteristic of M cells is their ability to uptake and transport particles across epithelial barriers through transcytosis. This distinctive feature is crucial in their role at barrier sites, allowing M cells to sample luminal antigens and initiate immune responses. First, the peculiar apical morphology of M cells allows for a less restrictive environment for antigen encounters when compared to the uniform densely packed microvilli of enterocytes (53, 56). This apical “antigen sink” can then be directly sampled by the M cells or by dendritic cells that can extend appendages through the epithelium and show a particularly close association with M cells (4, 31). Second, the basolateral pockets act as docking sites for immune cells and facilitate the movement of DCs and lymphocytes into the subepithelial dome (SED) (31, 58). These pockets typically contain B lymphocytes, though they do not appear to contribute to mucosal IgA antibody production and appear to be “mated” to their M cell for their short lives (59). The interaction between the basolateral pocket B cell and M cell also appears to be critical in licensing the transcytosis function of the M cell, as the absence of B cells or their interacting CD137 ligand leaves M cells with an approximated morphologic phenotype but lacking transcytosis function (60). This intricate cell complex allows for an efficient transition from antigen capture to immune cell interaction, enhancing the efficiency of mucosal immune responses. The M-cell pocket also serves to shorten the distance between the apical and basolateral surfaces, streamlining antigen transcytosis, which will be discussed in a later section. The unique combination of sparse apical microvilli and basolateral invaginations forms the architectural blueprint for M cells, finely tuned for efficient antigen handling and immune cell collaboration in the bustling environment of the mucosal interface.

The mechanisms underlying M-cell particle transport involve various cellular interactions and receptors, providing a gateway for both harmless particles and opportunistic pathogens. Aside from serving as a universal marker for M cells across species, GP2 functions as an uptake receptor for type I-piliated bacteria, such as *Escherichia coli* and *Salmonella typhimurium*, and as an entry point for botulinum toxin (61, 62). Other apical proteins that may mediate the specificity of M-cell transport include β1 integrin, cellular prion protein (PrP^c^), and IgA receptors (63–65). β1 integrin, in particular, mediates the internalization of *Yersinia enterocolitica* via invasin (66). Allograft inflammatory factor 1 (Aif1) has been implicated in this uptake pathway and may play a role in actin remodeling during the transcytotic process. Notably, Aif1 deficiency also affects the uptake of nanoparticles, suggesting a broader role in receptor-independent transcytosis (67). PCR analysis of toll-like receptor (TLR) transcripts in mice also found that TLR1, TLR2, TLR4, TLR8, and TLR9 were found to be highly expressed in M cells when compared to villous epithelium or FAE, and TLR2 has been implicated in proteasome-accelerated microparticle transport pointing to a potential role for these receptors in M-cell capture (68, 69).

Unfortunately, the array of uptake receptors for particles can complicate the notion of a specialized M-cell machinery, suggesting that multiple cellular elevators may be used by M cells for transcytosis. Many uptake receptors such as tight junction proteins may be useful for non-specific transcytosis mechanisms (e.g., Claudin-4 and coxsackie–adenovirus receptor) (70, 71). However, many uptake receptors, such as those mentioned previously, have been identified for their more specific role in binding and uptake of certain bacteria and viruses, which may have evolved adhesion receptors that simply take advantage of their access to M cells and, by binding, use available cellular machinery to hitch a ride across the epithelial barrier.

## 3 The non-classical “M cells”

In an attempt to begin a definition of M cells and classify any variants, we present a comparison of the myriad M-cell phenotypes that may not fit into a clear classical definition.

### 3.1 Villous M cells

The first deviation from the traditional GALT-associated M cell seems to be the villous M cell. Although present in the gut, they are not associated with an underlying lymphoid structure as are PPs, colonic patch, and isolated lymphoid follicle-associated M cells occurring instead, as the name suggests, on the villi. These cells can range from a few sparse cells to large-scale induction covering the tips of villi under sporadic or induced conditions (35, 72). Villous M cells also show a range of apical morphologies that seem to trend toward the usual scantly compact brush border of the PP M cell and also show a similar lectin binding pattern to UEA1 (73). Of note, the expression of M cell-associated genes is inconsistent (22), and interestingly, these cells were shown to still be present in various GALT-null models (35).

### 3.2 Type II taste cells

Taste sensation is orchestrated through the efforts of specialized taste cells dispersed within the taste buds of the oral cavity. These cells express a diverse array of taste receptors tailored to different taste qualities, including sweet, bitter, salty, sour, and umami. Once activated, taste cells, particularly type 2, initiate a signaling cascade resulting in the sensation of taste (74). Recently, Qin et al. published a study on the M cell-like characteristics of type 2 taste cells on the tongue (47).

Employing single-cell RNA sequencing, the gene expression profile of these cells was surveyed, finding a remarkable resemblance to that of M cells found in MALT. Furthermore, the administration of RANKL prompted the induction of an M cell-like gene expression signature in Type II taste cells including *Gp2*, *Marcksl1*, *Ccl9*, *Anxa5*, *Sgne1*, and *Spib*. The expression of these M cell-like properties in taste cells was contingent upon the presence of the transcription factor *Spib*, underscoring its regulatory role in orchestrating the cellular response to RANKL—a characteristic not dissimilar to the prototypical M cell (33, 47). The investigation further spotlighted the engagement of Type II taste cells in microbial transcytosis, a process akin to that observed in M cells. Through microbead uptake experiments, it was noted that WT but not SpiB knock-out (KO) taste cells were able to transcytose particles (47). This finding can potentially establish Type II as filling an M-cell niche at this mucosal site.

### 3.3 Thymic M cells

The thymus is one of two primary lymphoid tissues and has the important task of T-lymphocyte development and training. The key players in this process are cortical (cTECs) and medullary thymic epithelial cells (mTECs), which have critical roles in positive and negative selection, respectively. Specifically, we will focus on the medulla—the site of negative selection—where the mTEC compartment exists and through peripheral antigen presentation is able to aid in the training of thymocytes. This was thought to be done by the transcription factor AIRE (autoimmune regulator), which is able to induce tissue-specific antigen (TSA) expression on the thymic epithelia; however, a more complex mechanism may be at play (75).

Separately, two studies were able to identify peripheral cell mimetics that maintain many of the genetic (and potentially functional) hallmarks of the peripheral cells—among them, M cells. First, M cell-associated genes and mTEC subgroup included *Gp2*, *Pglyrp1*, *Tnfrsf11b*, *Tnfaip2*, and M-cell-specific chemokines *Ccl20*, *Ccl6*, *and Ccl9* as well as dependence on lineage-specific transcription factors SpiB and Sox8. Notably, thymic M cells are shown to have a “pocket-like” association with lymphocytes as well as a close association with CX3CR1+ APCs (48, 49). Givony et al. further expanded on these findings by establishing some of the retained M-cell features in this thymic analog such as bead uptake capabilities, B-cell recruitment and dependence through a CCL20-CCR6 signaling pattern, and induction of thymic IgA (48, 76, 77).

It has been suggested that these mimetics may proceed an Aire-stage mTEC, and through some set of stimuli, the expression of lineage-specific transcription factors yields the outcome of markedly peripheral-like cells in the thymus (78). For now, the driving mechanism for their induction is still unknown; however, these two methods of peripheral antigen expression seem to work together to maintain a diverse, non-self-reactive T-cell repertoire. Although in a tissue far from a mucosa, the thymic M cell may be employing its function and machinery differently in this organ and has been implicated in the maintenance of mTEC compartment cellularity (48), thus extending its reach into a potential influence on central tolerance.

## 4 To be or not to be: apical and basolateral cues for M-cell differentiation and organization

### 4.1 Basolateral (internal) cues

The discovery of M-cell mimetics in the thymus has now shown these cells to associate with the entire hierarchy of lymphoid tissues—primary, secondary, and tertiary—suggesting their broader role in immune training than previously suspected.

The prototypical M cell is associated with one of the many MALTs of the body. Whether it be the NALT of the airways, the GALT of the intestinal tract, or the tear duct-associated lymphoid tissue (TALT) of the eye (44), the association of these cells with the FAE has established a paradigm that has impacted the way these cells are studied. Now, we appreciate more fully that there are exceptions. In the lower airways, where lymphoid structures are not a constitutive element of the tissues, M cells were still found at the level of the trachea and bronchi (39, 40). Specifically, this showed that M cells can exist in the physiological lung. In the upper airways, M cells have been shown to exist independently of NALT, establishing that an underlying lymphoid structure is not crucial to the initial development of “respiratory M cells” (42). Type II taste cells also lack this feature as well as a basolateral pocket and an association with a germinal center containing lymphoid tissue (47). It was speculated that neighboring lymphoid tissue such as the lingual tonsils in humans and the NALT of mice could fill this role; however, as shown with “respiratory” and lower airway M cells, it may not be necessary.

Therefore, the question of whether these cells require an underlying organized lymphoid structure for differentiation cues is not as clear. Interestingly, the onus of M-cell fate determination is commonly placed on the cytokine/chemokine cocktail of the SED, offering a reason for the conceptual link between these cells and lymphoid structures. To this point, multiple signaling pathways in this region have been implicated in the initiation and maintenance of M-cell development. Most notably, the RANK-RANKL system is cited as being not only necessary but also sufficient to induce M-cell differentiation and gene expression in *in vitro* and *in vivo* studies. Notably, the key players in RANKL production are the stromal cells of the SED (37, 79). This is also the most utilized pathway to evaluate a suspected M cell; however, this induction system may still be reliant on the appropriate Lgr5+ stem cell, which may look different in varying tissues (20, 80). The RANK-RANKL pathway is upstream to the *SpiB*-lead functional development of GP2+ “mature” M cells. Of note, studies have shown that signaling mechanisms differ in their role in M-cell development. While SpiB is considered an established M-cell master regulator, Sato and colleagues reported that a potential SpiB-independent “M cell” subset may exist, which has a retained basolateral pocket structure with corresponding lymphocytes and the ability to transcytose certain bacteria (26). This is not to understate the importance of RANK-RANKL signaling on M-cell differentiation, as it seems conserved throughout many M cells in different tissues (**Table 1**). Instead, we bring attention to possible outliers that have not been fully characterized. In a broader context, it may be important to consider whether outlier phenotypes reflect the unique experimental conditions of a specific study, or whether the appearance of any novel “sport” outlier reflects biological variability that is simply suppressed under common conditions. Similarly, CD137–CD137L signaling is not necessary for the lineage commitment of PP or NALT M cells but can serve as a downstream signal for functional maturation, as CD137-deficient mice showed altered morphology and transcytosis abilities (60). Other signaling systems implicated in the development and organization of these cells include retinoic acid and Notch1 (81) (55, 82). However, whether these differentiation systems are a universal M-cell characteristic or reflect uniquely in specific tissues is left to be determined. Indeed, one widely accepted M-cell subtype is the villous M cell previously introduced. Compared to their FAE-associated counterparts, villous M cells have intriguingly been found to have differing induction systems where the induced villous subtype relies on TNFR2 signaling and not Ltβr as does the conventional PP M cell (83). In this way, an M-cell differentiation pattern is a product of independent, differentially regulated events determined by the host tissue.

Microfold mTECs may retain a dependence on RANK-RANKL signaling given that *ex vivo* studies on sorted thymic cells showed that RANKL stimulation induced *SpiB* expression (48). However, the source of RANKL could potentially deviate from the stromal cell-produced ligand of FAE-associated M cells in that thymocytes and invariant natural killer T cells (iNKTs) have shown to be RANKL producers in the thymus (84–86). Type II taste cells similarly respond to RANKL stimulation showing an increased proportion of M cell-like cells or increased M-cell gene expression in mouse and taste organoid studies, respectively (47). Combined, the retention of RANK dependence in thymic microfold mTECs and Type II taste cells may suggest that this system is an M-cell differentiation hallmark but, more significantly, may have different implications in these locations specific to each tissue.

### 4.2 Apical (external) cues

The immune system is not spatially restricted. This creates an interesting puzzle of figuring out where any specific cell is needed. White blood cells rely on networks of cytokines and chemokines that are able to call upon them. An epithelial cell, however, has access to multiple potential triggers including the outside (luminal) environment. The body is constantly interacting with particulates and microbes, necessitating high levels of surveillance, particularly at the epithelial boundaries. One would assume particle uptake mechanisms, as those described above would be found throughout every epithelialized tissue; however, a universal distribution of these uptake mechanisms is not observed. Instead, what is seen is a distinctive spatial distribution that turns out to be strikingly predictable where, in the case of M cells, this distribution shows a propensity to group together at predictable areas of the hosting tissue (**Figure 1**). Whether in the gut, lung, or tongue, they are reliably located in protected “nooks” that may serve as antigen reservoirs, and in examining the topography of these zones, they seem perfectly situated for optimal luminal sampling. The organization of Peyer’s patch is such that it is a sunken structure surrounded by the conventional villi. A cryptopatch, which is an abundant and rudimentary lymphoid structure in the intestine, can grow into a more developed isolated lymphoid follicle (ILF). This involves the recruitment, organization, and differentiation of various cells—including M cells—but we point to the larger modifications that take place where there are tissue-level structural changes that begin to resemble the conventional Peyer’s patch (87). In these ways, the different versions of GALT have adopted a somewhat predictable structure to funnel antigens to specific sites. Similarly, the documented patterning of M cells in the lungs of WT mice shows them grouped at branching points of the airways (39). Evaluations of particle accumulation have been shown in simulation studies of the lung where bifurcations seem to serve as deposition “hot spots” for particles in the micron to tens of microns size ranges (88, 89). On the tongue, the structure of the papillae, particularly the circumvallate papillae (CVP), has taste buds embedded into the entrenched sides of the larger structure. This may be an adaptation for efficient taste molecule sensing but also doubles as a microbial and antigen hub (47).

At the cellular level, this is furthered by the fact that M-cell antigen capture relies on apical physical properties. As mentioned above, these cells lack the prominent apical microvilli present on neighboring enterocytes; however, this compromise in surface area yields a gain in antigen capture capabilities—particularly bacterial. Examining the charge dynamics at the M-cell surface, the absent microvilli and altered surface glycoproteins result in a reduced repulsive charge, which in turn is able to draw microparticles closer (90, 91). Can a similar process apply to the tissue-level M-cell patterning? We will term this the M cell-dust bunny hypothesis based on the work of Bennett et al. on the electrostatics at the cell surface (89), where expanding on this analogy, we can appreciate these antigen accumulation areas as potential M-cell spawning points.

To assert further this “where there is a *need*, there is a way” argument for M-cell differentiation, we introduce the salmonid antigen-sampling cell and mosquito bare cell. Studies into the antigen-sampling capabilities of the salmonid intestine revealed a UEA1+ WGA− staining cell that colocalized with gold bovine serum albumin (BSA)-targeted particles in the base regions of the mucosal folds (92). Similarly, the bare cell exists in the midgut of *Aedes aegypti*, the yellow fever mosquito, and exhibits properties comparable to the M cell including a sparse apical surface and clustered organization throughout the midgut. Moreover, this cell seems to act as a preferential point of invasion by malarial parasites—a process reminiscent of the hijacking of M-cell receptors by microbes (93). Despite the presence of M-type cells in mucosal tissues of organisms evolutionarily distant from the mammalian systems, the existence of these cell types establishes that a universal biological need for surveillance at barriers can promote convergent outcomes even in some of the earliest forms of an immune system.

In combination, the identifiable patterning of these cells and the presence of analogs in more primitive systems suggest that M cell-like sampling mechanisms can appear as a potential response to an apical stimulus or where the need for antigen-sampling capabilities exists. However, the question remains: how are these antigen-sampling zones sensed? One possibility is that these accumulation foci are ideal environments for microbial colonization, so a role for pathogen-associated (PAMP) and danger-associated molecular pattern (DAMP) detection may be useful. Lipopolysaccharide (LPS), a component of gram-negative bacterial walls and a well-known TLR4 agonist, is implied to be able to induce bronchus-associated lymphoid tissue (BALT) or induced bronchus-associated lymphoid tissue, with associated M cells in the lung when neonatal mice are treated through to adulthood (40). Interestingly, MyD88, an adapter of TLR signaling, has also been implicated in M-cell differentiation potentially through an effect on RANK-RANKL-OPG signaling regulation (94). Another plausible mechanism may be the presence of physical sensors. Given that the mucosa is subject to outside physical forces, the detection of flow and pressure changes may be useful in determining where to establish immune hubs. Mechanosensors such as primary cilia exist in many tissues as monitors of sheer stress (95), and in the gut, piezo-mediated mechanosensation has more recently been described (96). These mechanisms are particularly relevant when considering the varying environments of the mucosa (e.g., bidirectional air flow in the lungs, peristalsis in the intestine, fluid properties, the 3D arrangement of tissues, and harmonic effects on basement membranes). Thus, it would be of value to determine whether these apical signals and physical forces could exert an influence on the epithelial and immune landscape.

The presence of seemingly functional M cells in the thymus is unique in that it is not a barrier tissue, and thus, the aforementioned sensing mechanisms may be deemed irrelevant. The thymus instead is a three-dimensional epithelialized structure in its mature form, and this geometry is crucial for proper function (97). However, in development, the TEC compartment exists as a stratified structure of epithelia from the third pharyngeal pouch endoderm that folds to become the thymus, so whether this time marks a critical point where these signals may influence the post-embryonic outcome is left to be explored. Moreover, one can argue that the thymus itself, although not a mucosal tissue, is an antigen reservoir—a characteristic necessary for proper immune training. Studies have described processes that deliver peripheral antigens to the thymus; thus, there may be antigen transfer among thymic M cells as part of an APC–M cell–B cell antigen transfer axis (11, 48, 98, 99). Whether these cells are in effect utilizing their antigen-sampling abilities within the domain of the thymus or have the potential to sample from circulation directly, as has been suggested by Givony et al. (48), is a topic of further study.

### 4.3 Signaling beyond antigen delivery

Having previously discussed the basolateral influences on M-cell differentiation, we will explore the lymphoid tissue–M cell cross talk with the assumption that is not exclusively unidirectional. Thus, could M cells be exerting an effect on their associated lymphoid tissue either by feeding an antigenic stimulus to underlying lymphoid cells or through direct cellular interactions? In the gut, conditional deletion of RANK in intestinal epithelium leads to delayed Peyer’s patch germinal center maturation and lamina propria IgA plasma cells, resulting in a downstream decrease in fecal secretory IgA (100). Moreover, the appearance of M cells at bifurcations of the lung correlates with observed patterning of induced BALT (iBALT) in these zones, and under multiple conditions, M cells and iBALT seemed to coexist, pointing to a fundamental link between the two (39, 40). In mice, conjunctiva-associated lymphoid tissue (CALT) may show a similar relationship where CALT in the mouse does not appear to be a constitutive element; however, when induced, the associated M cells show “typical” characteristics under the microscope (45). Whether the M cell or the CALT appears first has not been established, and physiological M cells have not been reported as they have been in the lung.

In the thymus, it is possible that M cells also play a role in the development and maintenance of this crucial lymphoid organ. Previously, RANK-RANKL signaling has also been implicated in thymic mTEC development (101), and SpiB seems to link the expression of mature mTEC markers, which include *tnfrsf11b* (OPG) (102). Indeed, SpiB−/− mice have been shown to have increased thymus cellularity and augmented mTEC compartments (48), so the self-regulation systems in play for M cells may have extended implications in the thymus. Whether control of mTEC cellularity is a direct or indirect effect through specific M-cell modulation or a product of parallel systems requires further study. Thus, the possibility that microfold mTECs may have an impact on thymic regulation may implicate these cells not only in T-cell tolerance in the periphery but also centrally (33). Overall, the relevance of these cells in the formation of these lymphoid tissues—whether primary, secondary, or tertiary—calls for studies to better and more mechanistically assess a role for M cells in development, maintenance, and organization.

## 5 M cells as a functional niche

Discussing the appearance of these cells in various tissues as well as the broader influences they may hold, the simplest question remains: what determines a *bona fide* M cell? Whether or not the cited “non-classical” cells can be considered true M cells is contingent on the definition that is used, and the paradigm set by the M cells of the GALT may only reflect the intestinal version of this cell. This variation highlights that it may be too simplistic to assume that a single marker can be universal in the identification of these cells. Previously, in establishing the presence of villous M cells, Jang and colleagues utilized a combinatorial method, taking into account lectin histochemistry of UEA1 binding, endocytic activity, apical morphology, and basolateral association with lymphocytes through a designated “pocket” (35). Strictly using these metrics, however, many of the previously discussed “M cells” would not qualify. Respiratory, tracheal, and Type II taste cells lack these intimate lymphocyte associations. In contrast, microfold mTECs show a close association with lymphocytes and antigen-presenting cells, endocytic capabilities, and UEA1 binding but lack a notable polarity to even assess apical adaptations as discussed previously (48, 49). More recently, while demonstrating the M cell-like characteristics of Type II taste cells, Qin et al. proposed a similar approach utilizing the more recently discovered M-cell markers such as *Spi-B*, *Sox8*, *GP2*, and *Tnfaip2* as a reliable detection strategy (47). Of note, respiratory M cells reportedly were not noted in the upper airways when immunoreactivity to SpiB and CCL9 was surveyed and less common SpiB-independent mechanisms may exist that still lead to an M cell-like phenotype, although further studies are needed to assert these cells as “approaching” M cell-like or as potentially valid novel cell phenotypes (26, 50).

**Figure 2:**
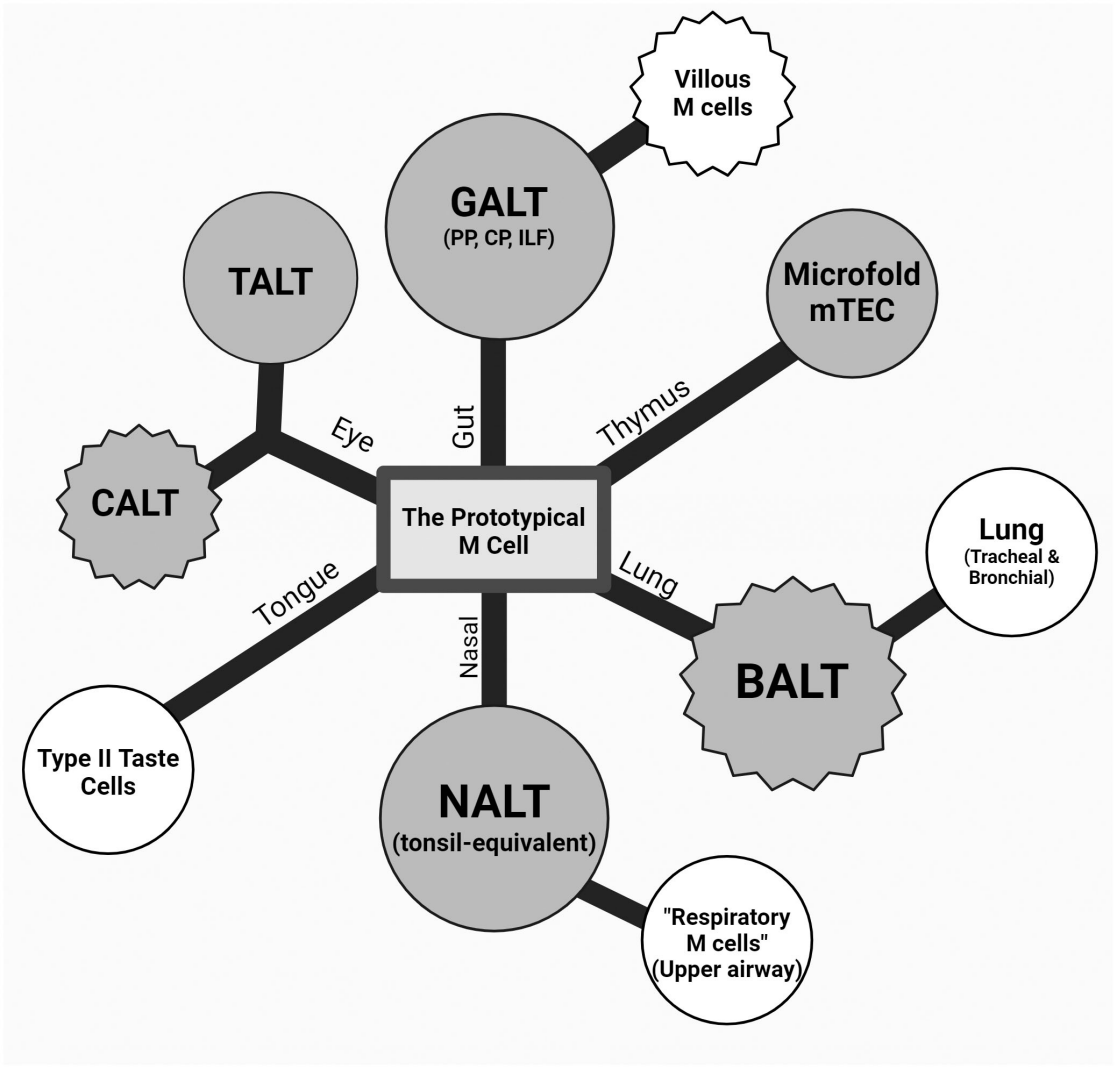
Theoretical dendrogram of the various M cells. A representative diagram of M cells from the intestinal, ocular, oral, and respiratory mucosa as well as the thymus (counterclockwise) with a center “prototypical M cell”. The first degree of separation mainly denotes the tissue and anatomical location, and the second branching point refers to a described cell type with a major difference in classical M-cell characteristics (i.e., association to lymphoid structures, varying presence of markers, and morphology). Nasal-associated lymphoid tissue (NALT) and respiratory M cells were drawn on the same branch due to this anatomical relationship and commonalities due to this (epithelium type, environment, etc.). Conjunctiva-associated lymphoid tissue (CALT) and tear duct-associated lymphoid tissue (TALT) are shown equidistant from the center, as they are both under the eye-associated lymphoid tissue (EALT) branch while capturing the difference between the seemingly constitutive TALT and inducible CALT in murine models. Circular and jagged outer edges were used to represent a constitutive and induced cell type, respectively, and color differences were used to show lymphoid tissue associations, where shaded M cells are those associated with a lymphoid structure, and those unshaded are not.

In defining the features that go into the definition of a differentiated M cell, we note that for any classification, there are going to be outliers or cells that marginally fit the core definition. Perhaps the essential error comes in allowing the initially identified cells to set the paradigm of what is an acceptable M cell. Comparing cells to one another, they all seem to lie on a spectrum of phenotypic and genotypic characteristics and can exhibit a “non-classical” nature dependent on the starting point or some “prototypical M cell” (**Figure 2**). The M cell, thus, is not a homogenous population; instead, we propose that M cells serve more as a functional niche, capable of being present in multiple environments. Further, we can speculate on whether these observed variations are a byproduct of the tissue origins of the cell, the initiating triggers, or some yet unexplored phenomenon.

## 6 M cells as an emergent property

Arguably, the cornerstone of M-cell identity is their antigen-sampling function; however, the way this is reflected in each cell’s floorplan may vary. To this point, we begin the argument that the specialized features of any M-cell population may be an emergent property of the tissue where it resides as well as the physical environment affecting the M cell and its function. First, M cells appear in tissues with very differing epithelial backgrounds even given the seemingly universal Lgr5+ endoderm prerequisite at the mucosa. The respiratory, intestinal, ocular, and oral mucosae serve very distinct functions and have very base level differences. For one, the type of epithelium, simple or stratified, ciliated or microvillus-containing, is determined by each location for optimal function; however, it seems that M cells are able to overcome these differences for a converging phenotype. Environmental differences have an influence as well. For example, M cells in the intestine overlying Peyer’s patches must emerge in an environment surrounded by fluid and various suspensions of particles of food and microbes, so its role in particle capture will be influenced by the mucus layer and the physical effects of peristalsis as well as the type and size of particles suspended in the intestinal lumen. By contrast, airway M cells exist at an air–liquid interface with rapidly reversing bidirectional flow, along with regions with boundaries between laminar and turbulent flow, and so the types and movement of particles available for capture will require functioning in a different physical environment. Indeed, each environmental circumstance may trigger a different type of response in potential M-cell progenitors.

From their discovery, early M cells were thought to be restricted to simple columnar epithelium overlying secondary lymphoid tissue in the gut, but now, their reach has expanded much further, and these conditions are apparently not so strict if at all. Therefore, we find ourselves chasing a moving target in defining these cells and constantly removing roadblocks in what we term an “M cell”. The idea of looking elsewhere has existed throughout many discussions. Oya et al., who characterized the TALT M cells, speculated on the existence of an M-cell phenotype for sampling in the human tonsil, vagina, and endometrium due to these tissues being covered in non-keratinized, stratified squamous epithelium.

We can broaden our search to other tissues where there is a need for surveillance and consider all locations based on the following criteria: the presence of an epithelial barrier, colonization by a microbiome, and an underlying network of cells to receive the “cargo” such as macrophages and dendritic cells. In this way, we speculate further on the presence of classical (or “non-classical”) M cells in the urogenital/reproductive tract. Moreover, even keratinized, stratified squamous epithelium may not be an absolute obstacle for M cells. In the skin, for example, structures such as sweat glands, sebaceous glands, and hair follicles may provide breaks in the keratinized barrier, offering potential openings suitable for the appearance of M cells.

As studies continue, the scarcity in numbers of these cells poses a challenge in discovery and characterization. One study sequencing the cells of the small intestine found that M cells required multiple levels of enrichment in order to meaningfully identify them as a cluster, going from approximately 0.01% of the initial sampling pool to approximately 0.4% when stratifying by FAE-enriched EpCAM+ cells of a tuft and M-cell reporter mouse (34). This seemingly holds true in the lung as well following a similar study (41), and therefore, the need to identify tissue-specific methods for enriching these cell populations will be crucial to further distinguish them through these kinds of studies.

## 7 M-cell triggers

Although the definition of a differentiated M cell is useful across tissues and physical locations, it does not capture the developmental or environmental triggers that induce M cells to form in specific tissues. For one, we can view the induction of an M cell at a tissue site as being created from convergent evolutionary forces serving a need for surveillance or monitoring of tissue barriers. However, the question is whether the same triggers act in all settings where M cells form. Based on the number of physical locations and associated molecular characteristics of M cells, we can imagine that M-cell triggering is a bit like a menu, where a requisite set of conditions, such as “one from column A, one from column B, etc.”, are cumulatively able to induce an M cell in that tissue. Thus, each tissue may give rise to M cells based on a different set of triggers. This all occurs within the historical context of the individual cells. Path dependence, or the idea that past decisions influence or constrain the outcome of a present state, has been explored at the organ and protein levels in biological systems (103). Here, we present this as a framework, at the cellular level, to view the varying flavors of M cells as a defined outcome of tissue-specific constraints and adaptations. Thus, when the proper initiating triggers are met at a particular developmental window, a *bona fide* M cell is born. Further, if this window is overcome due to overwhelming stimulus, is the result a transdifferentiated cell? Intriguingly, in the papillae, Type II taste cells are primarily recognized as sensory-specialized epithelial cells programmed to detect sweet, bitter, and umami flavors. The possible antigen-sampling and immune implications of these cells were not considered until broad transcriptomic surveys came into play. Therefore, then, did the taste-sensing function and immune-sensing function triggers happen to coincide in this cell at the right time and place, or did this taste cell come to fit some criteria for this immune niche? Overall, although we may not know what the set list of initiating triggers is for any single tissue, we can study tissue differences in microbial and mechanical sensing such as those mentioned above to aid in this understanding.

## 8 Closing comments

When faced with the challenges presented at the mucosal interface, biological systems exist to sense luminal contents hinting at their evolutionary benefit. At the tissue level, the structure and funneling of antigens seem crucial to proper immune function at the periphery, and this is only bolstered by cellular-level adaptations. The M cell is particularly well-adapted for its role in mucosal surveillance. Unfortunately, many reviews up till now have constrained their role to intestinal luminal sampling, but since their discovery in tissues such as the oral mucosa and thymus, this approach is not sufficient. In exploring the variety of tissues where M cells make an appearance, we sought to generalize the M cell essentially to their function, as a convergent phenotype relatively independent of specific developmental progenitor requirements. The transcytosis and particle uptake capabilities of these cells seem to place them at critical points at the epithelium, but understanding the site-specific triggers of each M-cell environment is crucial. For now, we are left to speculate on the potential mechanistic triggers of M-cell induction as we have mentioned above and further examine how this decision is informed by a cell’s history, specifically on the uptake machinery blueprint. We can acknowledge that M cell-like mechanisms exist throughout the evolutionary tree, and this may be a reason to consider that the underlying machinery may be far less complicated than initially thought.

Overall, our aim in this discussion is to develop a framework for the existence of M cells in different anatomical locations. The universal need for surveillance at barrier junctions seems to set the perfect stage for M cell-like qualities to arise, but the spectrum of tissues where they are found brings forth questions on what the cells are and what they do in a broader sense. Offering a definition for this cell, both molecular and functional, may only be a starting point, and we hope to reflect on what a more all-encompassing M-cell constitution may be. The epithelial landscape is unique in each tissue with distinct adapted characteristics and luminal environments, while a decision tree forms when faced with some requisite assortment of stimuli. Through this lens, we hope to have a better understanding and broader perspective of these cells in whatever novel tissue they may emerge.

## Author contributions

DD: Conceptualization, Formal analysis, Writing – original draft. DL: Conceptualization, Funding acquisition, Supervision, Writing – review & editing.

## Glossary

**Table:**

M cell	microfold cell
MALT	mucosa-associated lymphoid tissue
NALT	nasal-associated lymphoid tissue or nasopharynx-associated lymphoid tissue
GALT	gut-associated lymphoid tissue
iBALT	induced bronchus-associated lymphoid tissue
EALT	eye-associated lymphoid tissue
TALT	tear duct-associated lymphoid tissue
CALT	conjunctiva-associated lymphoid tissue
PP	Peyer’s patch
GAP	goblet cell-associated antigen passages
DC	dendritic cell
TEDs	transepithelial dendrites
FAE	follicle-associated epithelium
SED	subepithelial dome
ILF	isolated lymphoid follicle
UEA1	*Ulex europaeus* agglutinin 1
WGA	wheat germ agglutinin
GP2	glycoprotein 2
TLR	toll-like receptor
RANK	receptor activator of nuclear factor kappa-B
RANKL	receptor activator of nuclear factor kappa-B ligand
OPG	osteoprotegerin
Anxa5	annexin A5
Marcksl1	myristoylated alanine-rich protein kinase C substrate
Tnfaip2	TNF-α-induced protein 2
Sgne-1	secretory granule neuroendocrine protein 1
PGRP-S	peptidoglycan recognition protein
CCL	chemokine (C–C motif) ligand
AIF1	allograft inflammatory factor 1
PrPc	cellular prion protein
IgA	immunoglobulin A
CVP	circumvallate papillae
mTEC	medullary thymic epithelium cell
cTEC	cortical thymic epithelium cell
APC	antigen-presenting cell
PAMP	pathogen-associated molecular pattern
DAMP	danger-associated molecular pattern
KO	knock out
Ag	antigen
Ltβr	lymphotoxin-β receptor
TNFR2	tumor necrosis factor receptor 2
EpCAM	epithelial cellular adhesion molecule
